# Successful treatment of scleromyxedema with dupilumab: A case report

**DOI:** 10.1016/j.jdcr.2025.05.036

**Published:** 2025-06-21

**Authors:** Abdulelah Alghamdi, Abdulrahman Alfawzan, Khalid Aburas, Nedhal Ayoub, Mohammad Almohideb

**Affiliations:** a College of Medicine, King Saud bin Abdulaziz University for Health Sciences, Riyadh, Saudi Arabia; b King Abdullah International Medical Research Center, Riyadh, Saudi Arabia; c Division of Dermatology, Department of Medicine, King Abdulaziz Medical City, Riyadh, Saudi Arabia; d Prince Sultan Military Medical City (PSMMC), Riyadh, Saudi Arabia

**Keywords:** dupilumab, monoclonal gammopathy, primary mucinosis, scleromyxedema

## Introduction

Scleromyxedema (SMX) is a generalized form of lichen myxoedematosus that causes dermal mucin deposition, fibroblast proliferation, and occasional systemic involvement.^1^ The disease usually presents as 2-3 mm waxy, firm papules distributed over the hands, forearms, face, neck, and thighs while sparing the palms, scalp, and mucous membranes.^1^ It is usually linked to immunoglobulin G lambda monoclonal gammopathy.^1^^,^^2^ SMX affects middle-aged adults with normal thyroid function.^2^ Systemic manifestations may involve multiple organs, such as the musculoskeletal and cardiopulmonary systems, leading to significant morbidity and mortality.^2^ Diagnosis requires 3 out of 4 criteria. These include generalized papular and sclerodermoid lesions, monoclonal gammopathy, characteristic histopathology, and absence of thyroid disease.^2^ The pathophysiology of the disease is unclear; however, it is hypothesized that monoclonal gammopathy and several proinflammatory cytokines like interleukin (IL)-1, IL-6, and transforming growth factor beta are implicated.^2^ A recent study exploring circulating cytokines in SMX patients revealed that a chronically type 2 helper T cell-skewed T-cell response leading to abnormally high levels of IL-4, a profibrotic cytokine, is a main immunological hallmark of the disease.^3^ Due to SMX’s rarity, no definitive treatment guidelines exist. Yet, several agents showed variable responses in SMX, with intravenous immunoglobulin (IVIG) being the most widely used. Second-line agents like thalidomide and systemic corticosteroids have also been used, whether alone or in conjunction with IVIG.

## Case presentation

**Fig 1:**
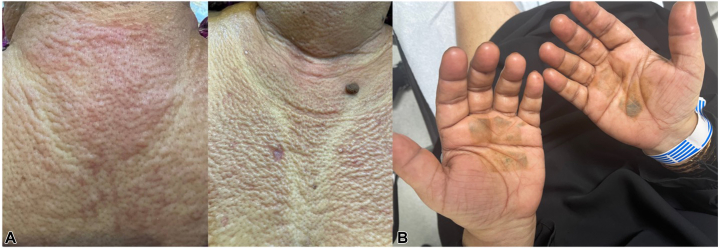
Images taken at initial presentation. Multiple erythematous and skin-colored waxy papules over the upper trunk **(A)**. Note the absence of palmar involvement, consistent with the typical distribution of scleromyxedema **(B)**. Pigmentation on the palms is due to henna application and is unrelated to the disease.

A healthy 52-year-old female presented with facial puffiness and itchy, indurated, erythematous papules linearly distributed over the upper back, chest, and upper limbs (Fig 1). Routine labs and a skin biopsy were performed. While awaiting results, prednisone 0.5 mg/kg, topical corticosteroids, and oral antihistamines were prescribed, but prednisone was stopped after 2 days.

**Fig 2:**
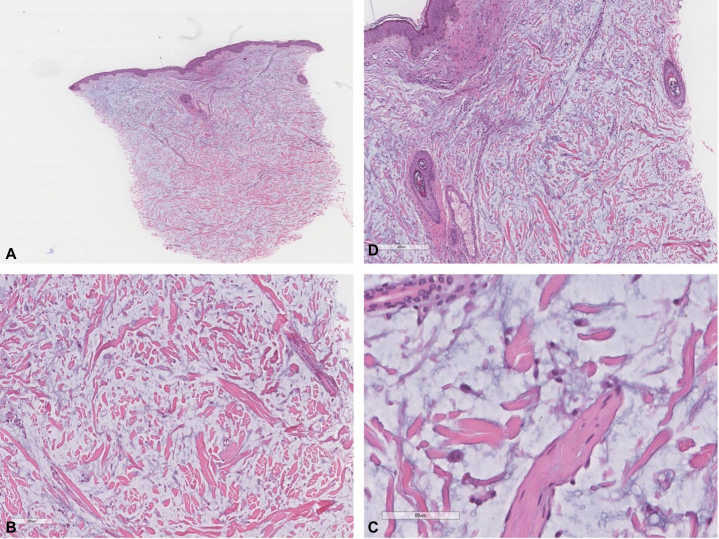
Histopathologic examination of the biopsy sample. Histopathologic features of scleromyxedema. **A**-**C,** Abundant mucin deposition within papillary and reticular dermis (**A-C,** Hematoxylin-eosin stain; original magnifications: **A,** 20×; **B,** 50×; **C,** 100×). **D,** The triad of fibroblast proliferation, thick collagen, and abundant mucin (Hematoxylin-eosin stain; original magnification: 200×).

Histopathologic findings showed dermal-based pathology, including mucin deposition along with fibroblast proliferation surrounding mucin with an unremarkable epidermis (Fig 2). On hematoxylin and eosin staining, mucin appeared as a pale blue-gray amorphous mass with increased fibroblasts showing prominent nucleoli and irregularly arranged thick collagen bundles. Alcain blue staining later confirmed mucin deposition, confirming SMX. Serum immunoglobulin analysis, serum immunofixation test, and electrophoresis revealed no evidence of monoclonal gammopathy. Other labs, including thyroid-stimulating hormone, were normal. A comprehensive systematic review, including imaging and laboratory tests, found no evidence of any other organ involvement.

**Fig 3:**
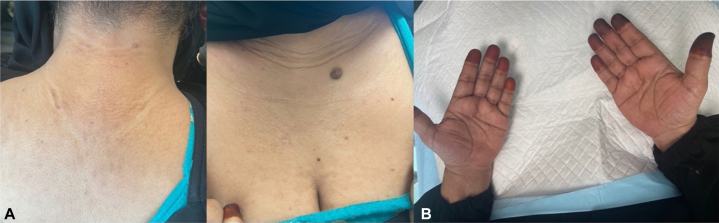
Images taken 12 weeks after initiation of dupilumab therapy, showing resolution of lesions. Complete resolution of upper trunk lesions **(A)** after 12 weeks of dupilumab. Note the continued absence of palmar involvement, consistent with the characteristic distribution of scleromyxedema **(B)**. Pigmentation on the fingertips is due to henna application and is not related to the disease.

**Fig 4:**
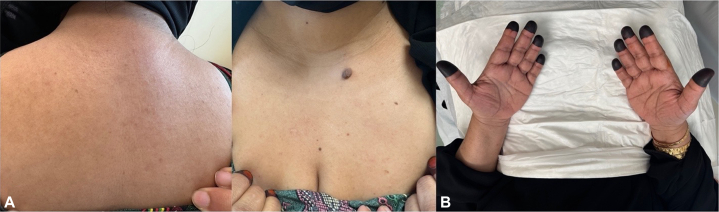
Images taken 48 weeks after initiation of dupilumab therapy, demonstrating sustained resolution of lesions. Chest and back at 12-month follow-up showing sustained clearance of popular lesions **(A)**. Palms at 12-month follow-up demonstrating lack of involvement **(B)**. Pigmentation on the fingertips is due to henna application and is not related to the disease.

Six weeks after the initial presentation, IVIG therapy (2 g/kg every 2 weeks) was initiated, and she experienced up to a 50% improvement. However, treatment was stopped because the patient, residing in a distant area, was unable to attend regular follow-ups, and symptoms returned to baseline severity. Alternative phototherapy was offered but was declined. Given the restriction to traditional treatments and based on emerging evidence for IL-4's implication in scleromyxedema pathogenesis, dupilumab (300 mg every other week), a monoclonal antibody for the IL-4 and IL-13 pathways, was initiated. After 12 weeks on dupilumab, she had total resolution of skin lesions, as confirmed in her follow-up dermatologic skin examination (Fig 3). At 12 months, she remains on dupilumab, free of symptoms and drug adverse effects (Fig 4). As she is in sustained remission, we plan to follow up on her response to prolonged therapy.

## Discussion

This article presents dupilumab use in SMX. Clinical data on SMX potential treatments is lacking. A systematic review undertaken by Haber R et al explored treatment options for SMX.^4^ In this study, IVIG was suggested as a first-line agent in treating SMX since it had proven to be beneficial, but certain limitations can pose a challenge. Thalidomide, used alone or as an adjunct to IVIG, has been proposed; however, side effects like neuropathy may limit its use. Other agents like melphalan, bortezomib, corticosteroids, and cyclosporine showed limited success in treatment due to either poor safety profiles and/or inconsistent responses.^4^

The pathophysiology behind scleromyxedema remains poorly understood, yet recent evidence indicates that an immune response skewed toward type 2 helper T cell with increased levels of IL-4 plays an important role.^3^ IL-4 and IL-13 are essential for stimulating fibroblasts to proliferate, inducing the production of glycosaminoglycans, and stimulating collagen deposition in tissues,^5^ which in turn contributes to mucin deposit buildup. Dupilumab is an anti–IL-4/IL-13 monoclonal antibody used extensively in treating atopic diseases. Given this biological overlap, we considered dupilumab as a rational therapeutic option for our patient. Her rapid response to dupilumab supports IL-4 inhibition as a potential treatment approach. Furthermore, the patient’s 12-month remission suggests that dupilumab may offer long-term disease control in select individuals. As little is known about scleromyxedema treatments over extended time frames, this provides an interesting observation regarding potential durability in inhibiting IL-4 in this disease.

The lack of monoclonal gammopathy, which is commonly observed in SMX, might partially explain our patient’s excellent response to dupilumab.^6^ Singh et al (2024) reported a dramatic response to low-dose prednisolone and thalidomide in a case of SMX without monoclonal gammopathy, suggesting that patients without monoclonal gammopathy experience a milder disease course and better response to immunomodulatory therapy.^7^ It remains unclear if this factor influenced our patient's response to dupilumab, and further research is necessary to determine the potential impact of this factor on treatment outcomes. Our patient's successful response supports the emerging evidence reported by Kalli F et al.^3^

To our knowledge, this is the first case report of successful SMX treatment with dupilumab. This agent can be an excellent choice for the management of SMX, given its relatively safe profile and ease of use. This report adds to the current SMX literature; however, more research is needed to determine the long-term effectiveness and safety of dupilumab in SMX patients. Dupilumab can be used as an alternative or adjuvant treatment for SMX, particularly when first-line agents are impractical or ineffective.

## Conclusion

SMX is a rare skin disease with extracutaneous manifestations. Its pathophysiology is poorly understood, and research evaluating potential therapeutic agents with respect to their safety and efficacy is lacking. In our patient, dupilumab was proved effective for SMX management; however, further studies are needed to better evaluate its efficacy and safety in patients with scleromyxedema.

## Conflicts of interest

None disclosed.
